# Comparing Video Head Impulse Testing in Patients With Acute Vestibular Dysfunction

**DOI:** 10.1097/ONO.0000000000000052

**Published:** 2024-04-12

**Authors:** Fumihiro Mochizuki, Yusuke Ito, Yoshiyuki Sasano, Erin Williams, Michael E. Hoffer, Manabu Komori, Izumi Koizuka

**Affiliations:** 1 Department of Otolaryngology, St. Marianna University School of Medicine, Kawasaki, Japan; 2 Department of Otolaryngology, University of Miami Miller School of Medicine, Miami, Florida.

**Keywords:** Vertical semicircular canal, Vestibular disorder, Video head impulse test

## Abstract

**Objective:**

The video head impulse test (v-HIT) can evaluate the function of all semicircular canals (SCCs) in a short period. In this work, we sought to compare v-HIT results among 2 commercially available devices, the ICS impulse (Otometrics, Denmark) (ICS) and Eye See Cam (Interacoustics, Denmark) (ESC), among individuals with unilateral vestibular disorders.

**Design:**

Retrospective study (n = 15).

**Setting:**

St. Marianna University School of Medicine Hospital.

**Patients:**

Fifteen patients with acute unilateral vestibular disorders.

**Intervention:**

Two v-HIT devices were conducted across patients to compare the results of the 2 models.

**Main Outcome Measures:**

Gain values and pathological saccades for each SCC were compared across the 2 models. Monothermal caloric testing was performed to compare alongside v-HIT gain values.

**Results:**

There was no difference between the 2 models for the evaluation of the horizontal SCCs. There was a significant negative correlation (ESC: r = −0.52, ICS: r = −0.53) between caloric testing and the gain values of the 2 models. In the vertical SCCs, the gain values of ESC were significantly higher than the gain values of ICS. Detection of catch-up saccades in vertical SCCs was similar across the 2 models.

**Conclusion:**

For the horizontal SCCs, there was no difference in test results between the 2 models. However, in the vertical SCCs, gain values were variable across the 2 devices. Larger scale studies are needed to develop normative ranges for the vertical canals.

The head impulse test (HIT) is performed by rapidly rotating the examinee’s head approximately 20° in the yaw plane and observing concurrent eye movements. Generally, the presence of catch-up saccades (CUS) determines the dysfunction of the horizontal semicircular canals (SCCs), which was first reported by Halmagyi and Curthoys (1). As one of the major tests of balance function, HIT is still widely used in otolaryngology and emergency medical care, but quantitative evaluation has been difficult because it is a subjective test method. Advances in technology have resulted in the development of the video head impulse test (v-HIT), which captures eye movements with lightweight goggles equipped with a high-speed video camera and gyro sensor, and then analyzes them on a laptop computer equipped with dedicated analysis software. v-HIT can evaluate the function of all SCCs, including the vertical SCCs as well as the horizontal SCCs (2).

v-HIT enables detailed regional diagnosis of vestibular dysfunction disease because the function of the superior and inferior vestibular nerves can be evaluated individually. A detailed assessment of vestibular function allows the treatment of vestibular disorders to be tailored to the individual; in fact, a systematic review demonstrated that while v-HIT is not a substitute for caloric testing, it has very high specificity (3).

There are several v-HIT models, each with its own characteristics. The ICS impulse (Otometrics, Denmark) (ICS) and Eye See Cam (Interacoustics, Denmark) (ESC) are the 2 most popular models. Presently, there are scattered reports demonstrating no significant differences in test results between the 2 models when limited to horizontal SCCs function (4–6). However, this has not held true for the vertical SCCs, where there have been significant differences in results (4–6). Here we report on 2 models of v-HIT performed on patients with unilateral vestibular disorders and compared the results of vertical SCCs as well as horizontal SCCs function.

## MATERIALS AND METHODS

### Participants

The participants are 15 individuals (4 males, 11 females; average age 58.5 ± 15 years) with acute unilateral vestibular disorders. Participants underwent an MRI of the brain, hearing tests, caloric tests, as well as 2 models of v-HIT testing. Details of the diseases of the 15 patients were as follows: 2 had Ramsey Hunt (13%), 7 had vestibular neuritis (47%), 5 had vestibular schwannoma (33%), and 1 had sudden hearing loss (7%).

This study was approved and conducted as a study by the Clinical Trials Subcommittee of St. Marianna University School of Medicine. The approval number is 2750. Both written and oral informed consent were collected before any study procedure and then documented appropriately in the medical record. All data and medical history obtained were anonymized so that individual patients could not be identified.

### Vestibular Function Tests

#### Caloric Test

Monothermal cold water caloric testing was stimulated by the minimal irrigation method (20°C, 5 mL) according to the standard provided by the Japan Society for Equilibrium Research (7). Eye movements were recorded using a video nystagmogram system or an electronystagmogram system, and the maximum slow-phase eye velocity was recorded in each ear stimulus. The percent difference in slow-phase velocity between the 2 sides was divided by their sum and defined as the canal paresis (CP). CP >25% qualified as unilateral vestibular loss, consistent with previous work (8).

#### Video Head Impulse Test

Table 1 summarizes the differences between the 2 models. The CUS were defined as being pathological only when all the criteria listed below were met (4,5): 1) must appear in more than 50% of all head impulses, 2) must have a minimum amplitude of at least 50% of the peak head velocity, 3) must appear in the opposite direction of the head turn, and 4) must occur from 100 ms following the onset of the head movement to 100 ms after the head movement has stopped.

**TABLE 1.** The characteristics of the ICS impulse and Eye See Cam

	ICS impulse	Eye See Cam
Calibrations	Eye movement	Eye and head movement
	(Horizontal)	(Horizontal and vertical)
Gain method	Area	Velocity regression
Camera position	Right side	Top side
Goggle parts	Foam material	Rubber
Sampling frequency	220 Hz	245 Hz
Eye position	Horizontal: center	Horizontal: center
	Vertical: side	Vertical: center
Head movement	Horizontal: yaw	Horizontal: yaw
	Vertical: pitch	Vertical: diagonally in the plane of LARP/RALP

LARP indicates left anterior-right posterior; RALP, right anterior left posterior.

Similarly, low gain was classified as being pathological when met the following criteria (4,5): 1) mean gain value below 0.8 for the horizontal SCCs with accompanying pathological saccades and 2) mean gain value below 0.7 for the vertical SCCs with accompanying pathological saccades. The 2 models of v-HIT were performed on the same day by the same examiner on the same participant. There was only one examiner in this study. The order of the model testing was randomized.

#### Eye See Cam

ESC has one detachable high-speed camera positioned on the upper side of the goggles to selectively capture eye movement, and the parts of the goggles to contact with the face are rubber. Subjects were seated 1.5 m from a wall with a fixation dot that served as the visual target. While the subject was asked to stare at the fixation dot, the examiner briefly and unpredictably rotated the subject’s head through an angle of 10°–20°. The head rotations were made in the horizontal, the left anterior-right posterior, and the right anterior left posterior planes. The ESC has 2 methods of measuring the vertical SCCs; the first method is to move the head obliquely in the direction of each vertical SCC without turning the head, and the second method is to rotate the head 45° yaw direction and then move the head vertically. In this study, only the first method was used to evaluate vertical SCCs function. The head impulses were repeated at least 10 times in each direction.

Participant eye and head movements were measured using video-oculography and inertial sensors. For the horizontal SCCs, there are 2 methods for calculating velocity gain values, first by the ratio of eye and head velocities at 60 ms after the onset of head rotation stimulation and secondly via velocity regression by comparing the slope of eye and head rotation velocity to the head acceleration (9).

**FIG. 1.**
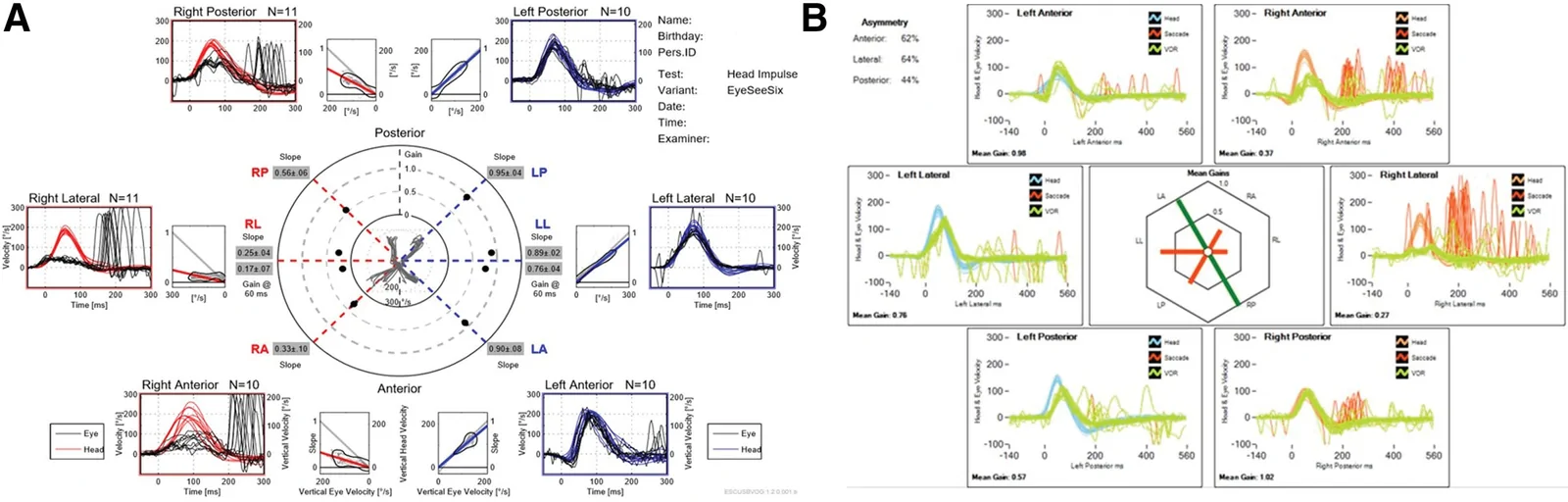
*A,* The Eye See Cam result from a patient with right vestibular neuritis (No. 6). All right semicircular canals show low gain and catch-up saccades. *B,* The ICS impulse result from a patient with right vestibular neuritis (No. 6). The test results show low gain and catch-up saccades in the right horizontal and anterior semicircular canals, and only catch-up saccades in the right posterior semicircular canal.

Since velocity regression is the only method for calculating gain values in the vertical SCCs, this study used the latter gain measurement, or gain value calculation with velocity regression. Figure 1A shows an example of participant data from the ESC.

#### ICS Impulse

ICS has a high-speed camera positioned on the right side of the goggles, capturing the movement of the right eye. The parts of the goggles in contact with the face are foam material. Subjects sit in the same way as in the ESC test, where subjects are asked to stare at the fixation dot then the examiner briefly and unpredictably rotates the subject’s head through an angle of 10°–20° to each SCC plane. Examination of the vertical SCCs requires moving the patient’s head 45° in the yaw plane and then in the pitch plane. The head impulses were repeated at least 15 times in each direction.

Gain values for the ICS are calculated using the area-under-the-curve (AUC) gain values. AUC gain values were calculated by averaging instantaneous gain values across the entire duration of the head impulse, not including any covert saccade (4,10). Figure 1B shows an example of participant data from ICS.

### Statistical Analysis

The paired *t* test and Pearson’s correlation coefficient test were used for the statistical analysis of this study along with Excel with add-in software Stacel4 (OMS, Tokyo, Japan). Significant differences were defined as *P* < 0.05.

## RESULTS

Table 2 shows the details of the participants and the results of the caloric test and v-HIT on the affected side. All participants had CP on the caloric test. Table 3 shows the gain of the 2 v-HIT models in all SCCs.

**TABLE 2.** The diagnosis, age, affected side, percent canal paralysis, and each v-HIT result for the affected side in our subjects

NO	Diagnosis	Age	Affected side	CP%	Horizontal SCC		Anterior SCC		Posterior SCC	
					ICS	ESC	ICS	ESC	ICS	ESC
1	Ramsay Hunt	41	Left	73.9	0.53/+	0.44/+	0.34/−	0.62/−	0.36/−	1.12/+
2	Ramsay Hunt	64	Right	79.1	0.44/+	0.42/+	0.42/+	1.14/+	0.36/−	0.86/−
3	Vestibular neuronitis	38	Left	28.4	0.46/+	0.51/+	0.32/−	0.66/−	0.50/−	0.39/−
4	Vestibular neuronitis	63	Left	100	0.36/+	0.22/+	0.96/−	0.21/−	0.78/−	1.04/−
5	Vestibular neuronitis	60	Left	100	0.21/+	0.03/+	0.20/+	0.86/+	0.53/−	1.36/−
6	Vestibular neuronitis	64	Right	100	0.27/+	0.25/+	0.37/+	0.33/+	1.02/+	0.56/+
7	Vestibular neuronitis	65	Right	85.0	0.55/+	0.53/+	0.78/−	0.98/−	0.77/−	1.04/−
8	Vestibular neuronitis	80	Right	100	0.31/+	0.17/+	0.31/+	0.35/+	0.37/+	0.26/+
9	Vestibular neuronitis	65	Left	100	0.47/+	0.49/+	0.5/−	0.6/−	0.36/+	0.25/+
10	Vestibular schwannoma	24	Left	72.4	0.6/+	0.65/+	1.17/−	1.04/−	0.76/+	0.77/+
11	Vestibular schwannoma	40	Left	36.5	0.5/+	0.37/+	0.14/−	0.73/−	0.04/−	0.14/+
12	Vestibular schwannoma	57	Left	100	0.09/+	0.26/+	0.52/−	1.15/−	0.25/+	0.47/+
13	Vestibular schwannoma	78	Left	70.0	0.52/+	0.69/+	0.53/−	1.05/−	0.4/+	0.86/+
14	Vestibular schwannoma	64	Right	100	0.41/+	0.25/+	0.27/−	0.49/+	0.26/+	0.40/+
15	Sudden deafness	76	Left	82.1	0.46/+	0.45/+	0.63/−	1.2/−	0.23/+	0.43/+

The v-HIT results showed the gain value and the presence of catch-up saccades.

CP indicates canal paresis; SCC, semicircular canal; v-HIT, video head impulse test.

**TABLE 3.** The results of gain values, paired *t* test, and Pearson’s correlation coefficient test by the 2 models in all semicircular canals

	ICS	ESC	*P*-value^a^	Correlation coefficient
Affected side horizontal SCC	0.41 ± 0.14	0.38 ± 0.18	0.31	r = 0.80, *P* < 0.01
Healthy side horizontal SCC	0.82 ± 0.11	0.85 ± 0.10	0.47	r = 0.26, *P* = 0.35
Affected side anterior SCC	0.50 ± 0.29	0.76 ± 0.33	<0.05	r = 0.20, *P* = 0.48
Healthy side anterior SCC	0.66 ± 0.22	1.11 ± 0.32	<0.01	r = −0.12, *P* = 0.65
Affected side posterior SCC	0.47 ± 0.26	0.66 ± 0.37	<0.05	r = 0.45, *P* = 0.09
Healthy side posterior SCC	0.60 ± 0.23	0.90 ± 0.17	<0.01	r = 0.12, *P* = 0.68

a Hypothesis testing was conducted to compare mean gain between ICS and ESC devices via paired *t* tests.

SCC indicates semicircular canal.

### Horizontal Semicircular Canals

**FIG. 2.**
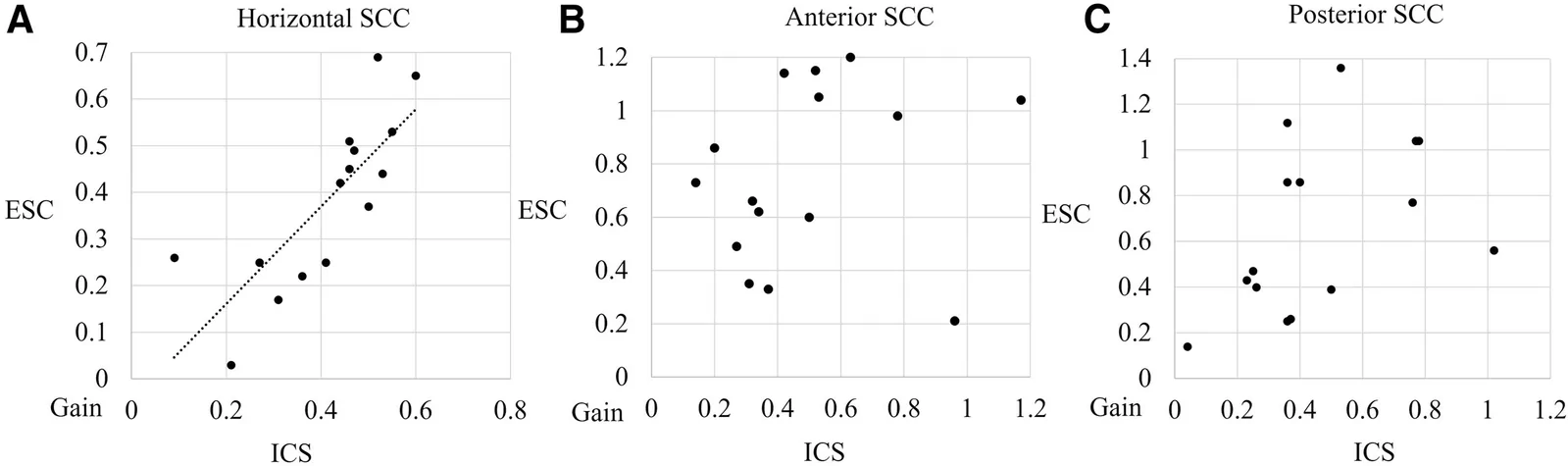
The correlation of the gain of the semicircular canals on the affected side with the 2 models (n = 15). The vertical axis of this graph is the gain of Eye See Cam and the horizontal axis is the gain of ICS impulse. *A,* There was a significant positive correlation between the 2 models on the horizontal semicircular canal (r = 0.80, *P* < 0.01). *B,* There was no significant correlation between the 2 models on the anterior semicircular canal (r = 0.20, *P* > 0.05). *C,* There was no significant correlation between the 2 models on the posterior semicircular canal (r = 0.45, *P* > 0.05).

The gain values of horizontal SCCs on the affected side across the 2 v-HIT models showed a significant positive correlation (r = 0.80; *P* < 0.01), with no significant difference in the gain values between the 2 models (*P* > 0.05) (Fig. 2A, 3A). The 2 models detected CUS in all subjects. There was a significant negative correlation between v-HIT and CP% for the ESC (r = −0.52, *P* < 0.05) and ICS (r = −0.53, *P* < 0.05), respectively. Conversely, the gain values of horizontal SCCs on the healthy side with the 2 v-HIT models were not significantly correlated (r = 0.25, *P* > 0.05), but there was no significant difference between the gain values of the 2 models (*P* > 0.05).

**FIG. 3.**
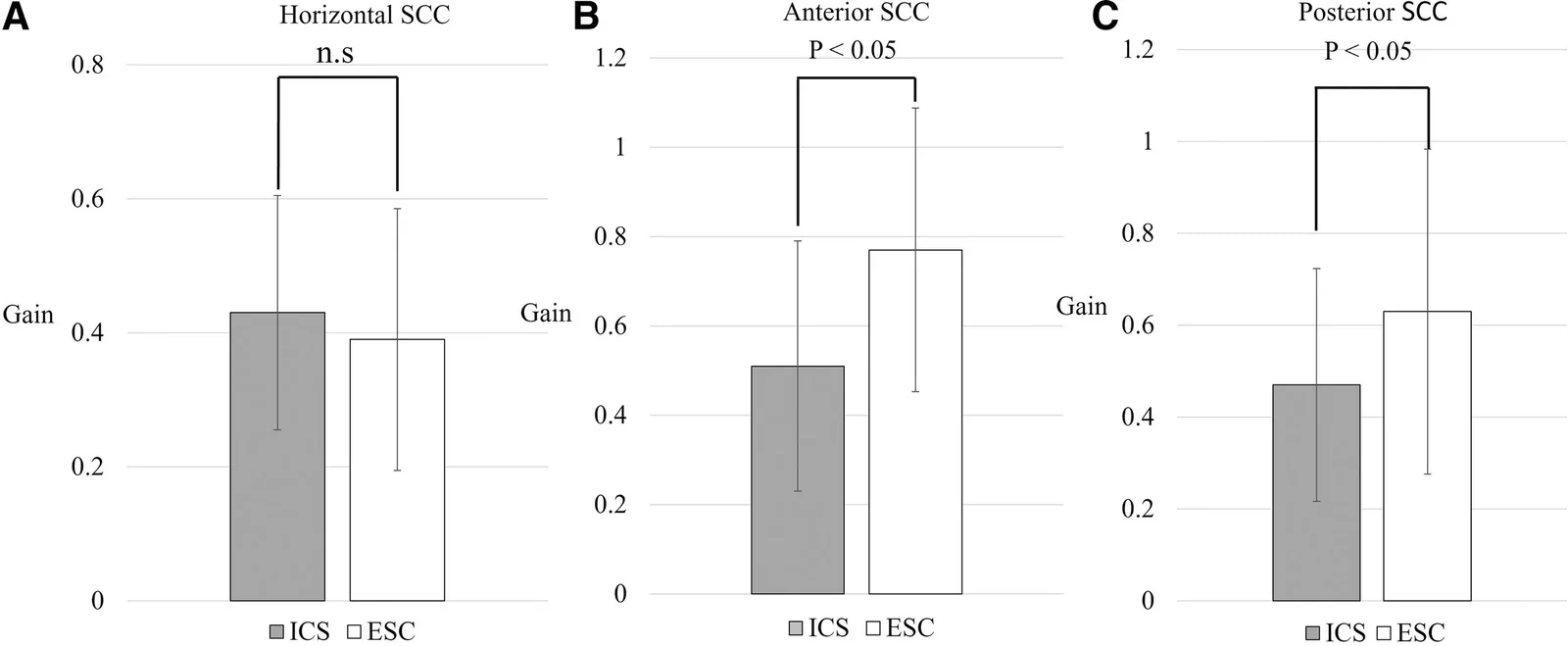
The comparison of the gain of the semicircular canals on the affected side with the 2 models (n = 15). *A,* There was no significant difference between the 2 models on the horizontal semicircular canal (*P* > 0.05). *B,* The gain of Eye See Cam was significantly higher than the gain of ICS impulse on the anterior semicircular canal (*P* < 0.05). *C,* The gain of Eye See Cam was significantly higher than the gain of ICS impulse on the posterior semicircular canal (*P* < 0.05).

### Anterior Semicircular Canals

The gain values of anterior SCCs on the affected side with the 2 v-HIT models were not significantly correlated (r = 0.20, *P* > 0.05), though the gain values of ESC were significantly higher than the gain values of ICS (*P* < 0.05) (Fig. 2B, 3B). CUS were detected in 4 cases with ESC and 5 cases with ICS. Again, the gain values of the anterior SCCs on the healthy side with the 2 v-HIT models were not significantly correlated (r = −0.12, *P* > 0.05) with significantly higher gain values with the ESC when compared to the ICS (*P* < 0.01).

### Posterior Semicircular Canals

The gain values of posterior SCCs on the affected side were not significantly correlated (r = 0.45, *P* > 0.05) across the 2 v-HIT models. ESC gain values were significantly higher than the gain values of ICS (*P* < 0.05) (Fig. 2C, 3C). CUS was detected in 10 cases with ESC and 8 cases with ICS. Eight cases detected CUS in both models. The gain values of posterior SCCs on the healthy side were not significantly correlated (r = 0.12, *P* > 0.05), though the gain values of ESC were significantly higher than the gain values of ICS (*P* < 0.01).

## DISCUSSION

This study compared the results of 2 different v-HIT devices with acute unilateral vestibular disorders. For the horizontal SCCs, there was little difference between the 2 models in gain values or in the detection of CUS. Common to the 2 models described in this study are a high-speed camera attached to a goggle headset, approximately similar sampling frequency, the placement of the eyes, and the operator movement of the head for the horizontal SCC test. Notably, the horizontal SCC testing conducted with the ESC and ICS showed no significant differences between the healthy and affected sides, but their gain values were not the same. Similarly, there was a correlation between the gain values of the 2 models on the affected side, but no significant correlation on the healthy side. Therefore, there may be a need to consider differences in models when assessing functionality based on gain values of the horizontal SCCs in studies. These small differences may be due to differences in the gain calculation methods of the 2 models and the materials of the goggles. However, when gain <0.8 and CUS detection were evaluated as horizontal SCC dysfunction, there was no difference in evaluation using both models. Although evaluation of the horizontal SCCs using these 2 models is useful, v-HIT is not sensitive in the chronic phase of horizontal SCC dysfunction and should be evaluated in combination with the caloric test (3). A previous study comparing horizontal SCC function in bilateral vestibulopathy patients with these 2 models, plus a model called Ulmer (Synapsys, Marseille, France), found that the gain values of Ulmer were significantly smaller than the 2 models (11). While Ulmer’s method of examining the horizontal SCCs is the same, the camera capturing the eye movements is placed in front of the subject and the sampling frequency of the camera is lower than in the ICS and ECS. From this, we can ascertain that it is important that the eye position and head movement during the test, the distance between the camera and goggles, and the sampling frequency of the camera must be consistent to avoid differences in the test results.

In the vertical SCCs, although most of the CUS detections were consistent between the 2 models, the gain values were significantly higher in ESC than in ICS. This is likely due to the difficulty in eliciting consistent head movements, and because recording the position of the eyes is variable between models. The differences between the 2 models in the vertical SCCs are further complicated by the anatomical location of the vertical SCCs, and the difference in magnitude of stimulation depending on how the head is moved. Ichijo reported that the anatomical location of the vertical SCCs is different among individuals based on the results of MRI studies of 50 patients (12). Due to individual differences in the vertical SCC, there may be differences in the input of obliquely and vertically rotated head rotation stimulation. In addition, head rotation adds tension to the sternocleidomastoid muscle, which causes the cervico-ocular reflex (COR) and the somatosensory input to the neck. There is a possibility that the stimulus to rotate the head 45° and then move the head in the pitch direction was greater than the stimulus of a straight head. Although increased compensation by COR has been reported in vestibular disorders with reduced VOR (13), in this study, the gain values of ESC, which are less affected by COR, were greater than those of ICS, which may be affected by COR, on both healthy and affected sides. As mentioned previously, in the vertical SCCs test of ESC, one method is to move the head obliquely, and the other is to move the head in the pitch direction (as in ICS). A comparative study conducted on healthy subjects found no difference in gain values obtained with these 2 methods (14). Therefore, differences due to the anatomical location of the SCCs and the force input from the cervical muscles to the vestibular system are not as significant in a quick input stimulus such as HIT, differences in head movement may not cause differences in vertical SCCs test. It is therefore suggested that the difference in test results between the 2 models in the vertical SCCs is due to differences in eye position rather than differences in head movement.

The VOR due to the vertical SCCs includes not only vertical movements of the eye but also torsional movements. Unfortunately, neither of the 2 models can measure the torsional movement of the eye (4). ICS inhibits this torsional movement by shifting the eye to the side. However, by shifting the eye sideways, the amplitude of the eye movement is smaller than in the horizontal plane. In ICS, the gain values of the vertical SCCs are known to be smaller than those of the horizontal SCCs, which are thought to be influenced by differences in the amplitude of eye movements (15). That is, although the amplitude of the eye is smaller in ICS, the stimulus applied may be greater than in ESC. By contrast, since there is a frontal eye position in the ESC, the amplitude of the eye is larger than in the ICS.

In vertical SCC, all patients with CUS detected in ICS also had CUS detected in ESC. Although there are reports of artifacts and examiner differences being influential in the detection of CUS (4,16), the examiner in this study was highly experienced, so these effects may be small. In our study, the concordance rate of CUS detection between the 2 models was high not only in the horizontal SCC but also in the vertical SCC. The detection of CUS was considered very important to reduce the differences between the 2 models in determining SCC dysfunction. Since the gain values of the 2 models in vertical SCCs are significantly different, each model should have its own definition of abnormal values for the vertical SCCs.

### Limitation

The study was conducted with a single examiner, among a small cohort of individuals with acute unilateral vestibular disorders. To determine normative and abnormal values for each model, this work should be replicated in large-scale studies with multiple examiners. In addition, vestibular rehabilitation has been reported to reduce the number and amplitude of overt CUS (17). Therefore, the detection rate of CUS is expected to be lower in chronic phase vestibular disorders. It is essential to investigate differences in the detection rate of CUS in the chronic versus acute phases with v-HIT.

## CONCLUSION

In this study, ICS and ESC were performed and compared in 15 patients with unilateral vestibular disorders. In the horizontal SCCs, there was no difference in the test results between the 2 models. In the vertical SCCs, although the gain values were significantly higher for ESC than for ICS, the 2 models were fairly similar with respect to the detection of CUS. Gain abnormalities should be defined in each model for the vertical SCC.

## FUNDING SOURCES

None declared.

## CONFLICT OF INTEREST STATEMENT

None declared.

## DATA AVAILABILITY STATEMENT

The datasets generated during and/or analyzed during the current study are not publicly available, but are available from the corresponding author on reasonable request.
